# Efficacy and tolerability of eslicarbazepine acetate as monotherapy in patients of newly diagnosed focal epilepsy

**DOI:** 10.1192/j.eurpsy.2021.2049

**Published:** 2021-08-13

**Authors:** S. Saxena, S. Singh

**Affiliations:** Psychiatry, TEERTHANKER MAHAVEER MEDICAL COLLEGE AND RESEARCH CENTRE, MORADABAD, India

## Abstract

**Introduction:**

Eslicarbazepine Acetate, a novel anti-epileptic drug has been approved as monotherapy in focal onset seizures, with/without secondary generalization in adults. Eslicarbazepine has many advantages over older anti-epileptic drugs and is useful in patients of new onset focal epilepsy.

**Objectives:**

Aim of our study was to determine the efficacy and safety of Eslicarbazepine Acetate, observe its well-tolerated use and monitor adverse effects in newly diagnosed patients of focal epilepsy.

**Methods:**

Study was done at Department of Psychiatry, Teerthanker Mahaveer University, Moradabad. A total of 30 newly diagnosed cases of focal epilepsy between 18-60 years of age were studied for 6 months, using a Semi-structured Interview and Liverpool Adverse Events Profile.

**Results:**

Majority of patients were males (58%), between 21-30 years. Patients with partial/focal seizures (63%) were more common than those of generalized seizures (37%). Majority of the participants had 1-2 episodes of focal seizures weekly(48%), while some had almost daily(32%). Majority were on Eslicarbazepine Acetate 800 mg in two divided doses daily (64%), while the others received 1200 mg in three divided doses(32%). The mean Liverpool Adverse Events Profile score initially was 28.34 ± 6.28 which significantly improved after 4 weeks treatment to 22.80 ± 4.35 (p < 0.05). The improvement in newly diagnosed focal seizures patients was significantly more than other patients (p < 0.05). No major side effects were observed.

**Conclusions:**

Eslicarbazepine Acetate as a monotherapy is effective in treating focal epilepsy. Better results of this drug are found in newly diagnosed focal epilepsy patients.

**Disclosure:**

No significant relationships.

